# Cultural Adaptation and Feasibility of an Inpatient Yoga Intervention for Patients Undergoing Hematopoietic Stem Cell Transplantation in Tanzania, India, and the United States: A Study Protocol

**DOI:** 10.1177/27536130261440941

**Published:** 2026-04-01

**Authors:** Smitha Mallaiah, Amanda L Olson, May Daher, Scherezade K. Mama, Santhosshi Narayanan, Nagarathna R, Raghavendra Rao, Sachin Jadhav, Nataraj KS, Vijaya Majumdar, Stella Rwezaula, Peter Muhoka, Manjunath NK, Lorenzo Cohen

**Affiliations:** 1 Department of Palliative, Rehabilitative and Integrative Medicine, The University of Texas MD Anderson Cancer Center, Houston, TX, USA; 2 560105 Department of Stem Cell Transplantation and Cellular Therapy, The University of Texas MD Anderson Cancer Center, Houston, TX, USA; 3 Department of Health Disparities Research, The University of Texas MD Anderson Cancer Center, Houston, TX, USA; 4 Division of Life Sciences, Swami Vivekananda Yoga Anusandhana Samsthana (S-VYASA), Bengaluru, India; 5 108089 Department of Integrative Medicine and Research, Health Care Global (HCG), Bengaluru, India; 6 Department of Hematology Oncology, Chiron Department of Hematology and BMT, Bangalore, India; 7 Department of Hematology Oncology and BMT, Health Care Global (HCG), Bengaluru, India; 8 Department of Hematology, Muhimbili National Hospital (MNH), Dar es Salaam, Tanzania

**Keywords:** yoga therapy, inpatient hospitalization, stem cell transplantation, study protocol, low and middle-income countries (LMIC), integrative medicine, cancer care

## Abstract

**Background:**

Cancer is a global health challenge that continues to rise worldwide. People with cancer in low- and middle-income countries (LMIC) have less access to integrative oncology programs than their counterparts in high-income countries. This is especially true of countries on the African continent, such as Tanzania. LMICs have a disproportionately higher cancer burden than their counterparts. Hematopoietic Stem Cell Transplantation (HSCT) requires extended hospitalization, including social isolation. While yoga is often recommended to help manage symptoms in oncology patients, its use has not been systematically evaluated among hospitalized patients or those undergoing HSCT. To address this gap, we designed a pilot study to culturally tailor and implement a yoga program for HSCT inpatients across diverse settings in Tanzania, India, and the United States.

**Methods:**

Five patients with hematological malignancy who underwent HSCT within the past year from each of the 3 centers will participate in semi-structured interviews to explore cultural norms, culturally appropriate language, and factors specific to yoga and psychosocial support. After any modifications to the program, 15 patients from each center will participate in the yoga program 2 weeks before HSCT and for the first 30 days after HSCT via in-person, telehealth (Zoom), or in combination. We will declare the intervention feasible if (a) at least 50% of patients complete a minimum of 6 of the 12 YT sessions and (b) at least 60% of patients complete follow-up measures at 2 months after transplant.

**Conclusion:**

This trial will provide important information regarding the cultural sensitivity and feasibility of an inpatient yoga program provided during hospitalization for 3 different centers. This data is necessary before embarking on a multicenter randomized clinical trial.

## Background

Cancer is a global health challenge that knows no borders. Low- and middle-income countries (LMIC) have a disproportionate cancer burden compared to high-income countries. LMICs represent 80% of the global cancer burden but receive only 5% of global cancer-related funding, making it increasingly difficult for them to deliver high-quality cancer care to their populations.^1,2^ Patients in LMICs face significant disparities in access to both conventional cancer care and mind-body integrative oncology services compared to those in high-income countries. This is especially true of countries on the African continent, such as Tanzania.^3^ However, people with cancer experience similar challenges to their quality of life (QOL), regardless of where they live. Patients experience fear of disease progression, recurrence, and death, all of which cause significant distress.^4^ People with cancer also experience a decline in their QOL due to the side effects of treatment.^5,6^ Research suggests that mind-body programs may help patients cope with the acute side effects of treatment and improve QOL during and after treatment.^7,8^

Extended periods of hospitalization can lead to fatigue, sleep disturbance, depressive symptoms, and functional decline in people with cancer.^9^ Hematopoietic Stem Cell Transplantation (HSCT) is 1 such procedure requiring extended hospitalization. In HSCT, diseased or cancerous bone marrow is replaced with healthy stem cells. Though HSCT is a life-saving intervention, it is associated with a multitude of physical and psychological sequelae that patients continue to experience long after their HSCT. Acute side effects of HSCT include anorexia, skin, eye, and oral problems, fatigue, anxiety, depression, and insomnia.^10,11^ Late complications can consist of neuropsychological symptoms such as depression, anxiety, posttraumatic stress disorder, neurocognitive deficits, cardiopulmonary and endocrine problems, and increased risk of infections and secondary cancers.^11^ Therefore, interventions are necessary to support patients undergoing HSCT during hospitalization and recovery.

Yoga is an ancient practice from India that integrates various postures of movement, breath work, mindful awareness, and lifestyle to support balance in body, mind, and spirit. Yoga Therapy (YT) is an evidence-based mind-body practice that can be used to support various medical conditions including across the cancer continuum from diagnosis to survivorship, as well as alongside end-of-life care.^12^ The International Association of Yoga Therapists defines Yoga therapy as “The professional application of the principles and practices of yoga to promote health and well-being within a therapeutic relationship that includes personalized assessment, goal setting, lifestyle management, and yoga practices for individuals or small groups.”^13^ Yoga is increasingly being utilized due to a better understanding of how chronic stress worsens cancer outcomes and the extensive evidence suggesting that it improves QOL, physiologic outcomes, and biological processes.^14^ A review of 29 clinical trials found that low-intensity forms of yoga are safe and effective for relieving sleep disruption, nausea, fatigue, pain, psychosocial distress, and musculoskeletal symptoms,^15^ which are often experienced by patients during HSCT. Yoga may also reduce cognitive impairment associated with cancer treatment.^15^ Yoga has been associated with decreased cortisol response, increased cell-mediated immunity, and decreased inflammatory biomarkers,^16-18^ all of which may lead to improved outcomes for those undergoing HSCT. The National Comprehensive Cancer Network (NCCN) has endorsed yoga in managing cancer-related fatigue and anticipatory nausea/vomiting and suggests “considering” yoga for distress (anxiety/depression/trauma), pain, cognitive function, and menopausal symptoms.^19^

No comparable studies have investigated yoga’s efficacy in an inpatient setting or with adult patients undergoing HSCT. There are also no culturally-tailored mind-body programs to support people with cancer in African countries. Moreover, yoga has predominantly been studied in group-based settings, which can be challenging for patients undergoing HSCT to attend, as they are often isolated to minimize infection risk due to low white blood cell counts. The extended hospitalization, social isolation, and the physical and psychological symptom burden commonly experienced by patients undergoing HSCT create an ideal opportunity to engage in yoga during their hospital stay. Also, YT is clinically indicated in this setting due to the low cost, compatibility with medical treatments, extensive evidence base in an outpatient setting, ability to individualize treatment, and minimal side effects.^20,21^

Although yoga has been studied and is more widely available in the US and India, the same is not true in Tanzania^22^; There are currently no culturally tailored mind–body programs to support people with cancer in many African countries. This gap underscores the need for culturally tailored, low-cost mind–body interventions that align with local beliefs and community contexts and are feasible for implementation in resource-constrained settings across Africa.^23^ Even though there have been attempts to deliver yoga in pediatric HSCT populations,^24-26^ no clinical trials have been developed providing inpatient yoga to adult patients undergoing HSCT and examining the effects across multiple countries within 1 multicenter/country trial.

The proposed multicenter involves 3 primary institutions: The University of Texas MD Anderson Cancer Center in Houston, USA, Swami Vivekananda Yoga Anusandhana Samsthana (S-VYASA) in Bengaluru, India, Healthcare Global Enterprises (HCG), headquartered in Bengaluru, India with 24 medical centers across the country and 7 performing HSCTs, and Muhimbili National Hospital (MNH), a government-run hospital in Dar es Salaam, Tanzania. MD Anderson has a longstanding collaboration with S-VYASA in developing and implementing clinical trials of yoga in oncology settings. Similarly, S-VYASA collaborates with HCG, where they oversee the clinical delivery of yoga programs and integrative oncology. In 2021, HCG started working with MNH specifically to develop and oversee its HSCT program. Every 3-4 months, a team from HCG travels to MNH for a month, where they perform HSCTs.

The aim of this pilot, multicenter study is to develop and culturally tailor an inpatient yoga therapy program for adults undergoing HSCT in the United States, India, and Tanzania, and to evaluate its feasibility, safety, and preliminary effectiveness across these settings.

## Study Design

**Figure 1.**
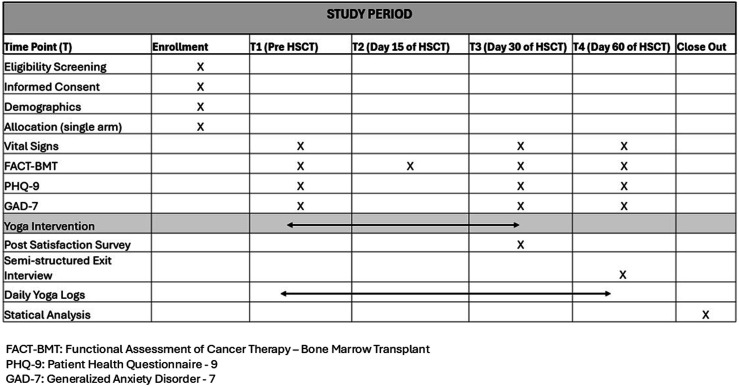
SPIRIT figure for the schedule of enrollment as recommended by the 2013 SPIRIT statement

This is a single-arm, prospective, multicenter pilot study designed to evaluate feasibility, conducted in accordance with the 2023 SPIRIT (Standard Protocol Items: Recommendations for Interventional Trials) Statement, which provides guidance for the development and reporting of interventional trial protocols^27^ (Figure 1). This protocol has been approved by the Institutional Review Board at MD Anderson Cancer Center (protocol # 2022-0785) and the IRBs of HCG and MNH. This study will follow a stepwise approach to culturally tailor the YT program for HSCT patients, divided into 2 phases: developmental (Aim 1) and optimization (Aim 2). For Aim 1, the program will be refined using feedback from semi-structured interviews with 5 patients from each of the 3 centers. The process will continue until thematic saturation is reached. Aim 2 will involve the initial testing of the YT program at each clinical center, using feedback from patients to optimize the intervention before a larger multicenter randomized controlled trial. The primary aim for Aim 2 is feasibility, as our small sample size is not powered to detect statistical significance. However, collection of quantitative questionnaires and conducting exit interviews is part of the feasibility for a future randomized clinical trial.

### Infrastructure and Program Development (Aim 1)

Before initiating the study, Standard Operating Procedures for data collection and YT delivery will be developed, and regulatory binders and case-report forms will be standardized. For Aim 1, at least 5 patients per site who have recently completed HSCT will complete 1 semi-structured, interview to inform the development of the YT program, using the semi structured interview guide (Supplemental Document). To participate in Aim 1, patients will be over the age of 18, able to speak English, Hindi, or Swahili, and capable of providing written informed consent. We aim for balanced representation of men and women (all participants are eligible regardless of gender identity) and allogeneic and autologous transplant recipients.

The collaborative team will develop the semi-structured interview guide, drawing on the expertise of MD Anderson, S-VYASA, and HCG, with input from MNH on cultural and logistical considerations. Individual interviews will be conducted with at least 5 patients per site, or until thematic saturation (referring to the point at which no new themes, concepts, or insights emerge from additional interviews).

The interview guide, developed using the Adaptome Framework, explores how health interventions are adapted across populations and settings while preserving effectiveness.^28,29^ It covers reasons for adaptation, service settings, target audience needs, delivery modes, cultural alignment, while retaining core components. Previous studies have used this framework to document how interventions are culturally adapted while preserving core components, including shifts in delivery style and language to enhance cultural fit.^30,31^

Semi-structured interviews lasting 45-60 minutes will inform a culturally sensitive yoga therapy intervention for HSCT patients. They will explore patients’ transplant experiences, symptom burden, coping strategies, and attitudes toward yoga and meditation. Participants will review the proposed yoga program and provide feedback on its utilization during the first 30 days post-transplant. Preferences regarding session frequency, duration, timing, and delivery mode (in-person or Zoom) will be assessed, along with cultural considerations such as instructor characteristics, representation, language, and accessibility. A research coordinator or nurse from MD Anderson will oversee interviews at MNH.

### Optimization and Initial Testing (Aim 2)

Each of the 3 centers will recruit 15 patients prior to HSCT hospitalization. The YT program will begin 2 weeks before transplant and continue for 30 days following HSCT, offering 2-3 yoga sessions per week, for up to 12 sessions. Feasibility will be measured by patient retention (completing follow-up at 2 months post-transplant) and adherence (completing 50% of sessions). Yoga will be delivered in-person, via Zoom, or a combination. Assessments will occur at baseline, 15 days post-transplant, and 1- and 2-month post-transplant. The 1- and 2-month time points represent post-intervention follow-up assessments. Satisfaction surveys and semi-structured exit interviews will be conducted.

### Patients

**Inclusion Criteria:** Patients with cancer scheduled to undergo an autologous or allogeneic HSCT at MD Anderson, HCG, or MNH; age 18 or older; English, Hindi, or Swahili speaking; able to provide written informed consent; and willing to follow protocol requirements.

**Exclusion Criteria:** Extreme mobility issues that preclude participating in the YT as determined by clinical observation and medical history; major thought disorders such as schizophrenia or uncontrolled bipolar disorder, assessed through medical history; and A Hematopoietic Cell Transplantation Comorbidity Index (HCT-CI) comorbidity score of 3 or higher, excluding cancer diagnoses. Participants with current, regular yoga practice within the past year will be excluded.

### Study Procedures

Eligible patients will be recruited 1-3 weeks before hospitalization for HSCT. The study coordinator will obtain consent, as outlined in the Informed Consent Document (ICD, Supplemental Document). Baseline data will be collected at least 1 week before admission, either in person or virtually. Each of the 3 centers will recruit 15 patients, aiming for equal representation of allogeneic and autologous transplant recipients. The goal is to assess the feasibility and initial efficacy of the YT program, with patient feedback used to refine it before a larger RCT.

### Intervention

The YT program can begin 2 weeks prior to transplant and continue during the first 30 days of hospitalization, for up to 12 sessions. Yoga classes will be held up to 3 times a week, with each session lasting 60 mins or adjusted based on patient tolerance. Sessions will be delivered in-person, via videoconferencing (Zoom), or a combination by Certified Yoga Therapists (C-IAYT’s) with at least 5 years’ experience in working with a cancer population. For online or tele-visits, we confirm the patient’s current location before the session begins and review the emergency response plan, including ensuring that a caregiver is present in the room so appropriate support can be activated if needed. Patients will be provided with a yoga mat and audio and video recordings for self-practice. After this period, they will practice independently for an additional month, either at the hospital or at home, to assess adherence post-instruction. All sessions will be recorded for review, using a standardized checklist for ongoing feedback. Recordings will be deleted within 10 years of publication.

Feedback from Aim 1 will inform the development of Aim 2 practices for patients. The final practice list will be finalized based on input from yoga therapists, HSCT oncologists, other medical providers, and researchers on the study team. The YT program will include 4 main components: (1) joint loosening and stretching, (2) yoga postures and shavasana (relaxation), (3) pranayama/breathing techniques, and (4) meditation. We will use an adaptable yoga therapy protocol designed to meet the evolving needs of this highly symptomatic population. Our C-IAYTs will draw from a range of appropriate practices based on individual patient needs. The protocol emphasizes gentle, low-intensity yoga practices tailored to the needs of hospitalized HSCT patients. Postures or techniques that could pose undue strain (inversions, deep twists), affect balance, or interfere with medical safety will be avoided, particularly during the early post-transplant period. Practices are adapted to each patient’s condition, ensuring safety and feasibility within the hospital setting. Self-reported yoga logs will be collected for 30 days after instruction. Completion of Aim 1 will inform the finalization of practice components for Aim 2. This intervention description aligns with the CLARIFY 2021 guidelines for transparent reporting of yoga research.^32^

### Study Measures

Patients will complete assessments through electronic questionnaires, with a paper-and-pencil option available. All responses will be collected and stored in the REDCap database, with paper responses manually entered by the research team. A clinical research coordinator (MD Anderson and HCG) will oversee data collection for self-report measures, conduct exit interviews, and extract basic information from the medical record, including gender, age, weight, height, medical history, cancer stage, HSCT procedure, biometric data during participation in the study, progress notes, and all labs pertinent to treatment.

Questionnaires will be collected at baseline, which is pre-HSCT (T1), 15 days ± 5 days (T2; just FACT-BMT), 1 month after transplant ± 7 days (T3), and 2 months after transplant ± 7 days (T4). To minimize the burden, patients will complete 3 standard oncology assessments. Validated Hindi versions are available; Swahili-speaking participants will receive oral translation by trained staff, as written Swahili versions are unavailable. Below is the list of questionnaires for AIM 2.

1. Demographic questionnaire assessing background factors.
2. FACT-BMT^33,34^ The subscale, combined with the FACT, is a 47-item valid and reliable measure of 5 dimensions of quality of life in bone marrow transplant patients.
3. Patient Health Questionnaire-8 (PHQ-8)^35^ The PHQ-8 is an 8-item self-report tool assessing depressive symptoms over the past 2 weeks, scored from 0 to 24, with higher scores indicating greater distress and impairment.
4. Generalized Anxiety Disorder-7 (GAD-7).^36^ The GAD-7 is a 7-item self-report tool used to assess anxiety symptoms over the past 2 weeks, scored from 0 to 21, with higher scores indicating greater distress and impairment.
5. Patients will complete a satisfaction survey at T3.
6. Weekly yoga logs: Starting after the first week of yoga instruction, Patients will keep yoga logs to indicate when they practice and a visual analogue scale of 0-10 of before and after feelings, including anxiety, relaxation, pain, nausea, and fatigue. The logs will be completed weekly through Day 60 (T4).
7. Exit interviews: After participating in the intervention program, all patients will complete a one-time exit interview. The interview will be recorded using both audio and video and will ask patients about their experience in the study, including what they liked, what they didn’t, and any suggestions for improving the program for future patients. The interview will be conducted either in person or via video conference and de-identified for analysis, following the Exit Interview Guide (Supplemental Document).

All adverse events (AEs) and serious adverse events (SAEs) will be systematically collected, documented in the REDCap database, assessed, and reported by Good Clinical Practice (GCP) guidelines and applicable regulatory requirements.

## Data Confidentiality Procedures

Patient identifiers (eg, medical record number, cancer diagnosis dates, treatment status, hospitalizations, and death) will be securely stored. Each Patient will be assigned a study number, and personal data will not be disclosed. The key linking identities to study numbers will be securely held by the investigator. Names, email addresses, and phone numbers will be collected to distribute the invitation letter and REDCap survey link. For Patients in Tanzania and India, contact will be primarily via text message through Twilio (https://www.twilio.com/), due to limited email access. In the U.S., email will be the primary mode, with text as a backup if preferred. Survey responses will be de-identified using a unique study code. Only the data manager will know who responded but will not have access to their responses. Access to the final trial dataset will be restricted to the principal investigator and designated study team members involved in data analysis. All individuals granted access will be required to adhere to data confidentiality agreements.

### Data Analysis

Qualitative Analysis: Semi-structured interviews from Aim 1 and Aim 2 will be audio- and video-recorded, transcribed verbatim, de-identified, and analyzed using qualitative methods. Two independent reviewers will transcribe each interview separately, and a third reviewer will reconcile differences into a final version. All transcripts will be reviewed for accuracy and stored securely prior to thematic analysis. ATLAS.ti or similar software will be used if needed.

A thematic approach will guide analysis, using an inductive (“bottom-up”) coding process to identify emergent themes.^37^ Anticipated “top-down” theory- or literature-based themes may also be explored. Preliminary themes will be compiled, refined through team discussion, and finalized by removing duplicates and merging overlapping areas.

Quantitative Analysis: Quantitative data will be summarized using descriptive statistics (mean, SD, median, range for continuous variables; frequency and proportion for categorical variables). Distribution will be assessed via visual methods and the Kolmogorov-Smirnov test. Changes in psychosocial outcomes (FACT, PHQ, GAD) from baseline to follow-up (T2, T3, T4) will be analyzed using paired t-tests or Wilcoxon signed-rank tests, depending on normality, with results interpreted for hypothesis generation only. Clinical cut-offs for anxiety and depression will be examined, and differences between autologous and allogeneic transplant recipients explored. Associations between outcomes and yoga adherence (eg, number of sessions) will be evaluated using Pearson correlations, chi-squared, or Fisher’s exact tests, as appropriate. Baseline HCT comorbidity scores will also be assessed, though causal inferences will be avoided due to potential reverse associations. As outcomes are expected to decline over time due to treatment burden, data will be interpreted descriptively rather than to test yoga’s buffering effect.

**Sample size determination:** For Aim 1, up to 5 semi-structured interviews will be conducted with past HSCT patients at each of the 3 sites, or until thematic saturation is reached. For Aim 2, 15 patients per site will be recruited.

**Feasibility and Pre-Post Tests:** Feasibility will be defined as at least 50% of participants completing 50% of the 12 YT sessions, and 60% completing follow-up assessments at T4. The sample size (n = 45 across 3 sites) supports thematic saturation and allows reasonable precision for feasibility estimates.^38^ For example, with 45 Patients, a 95% confidence interval for an expected 50% adherence rate will extend ±14.6%, and for a 60% follow-up rate, ±14.3%. As a developmental feasibility study without a control group, formal power calculations and hypothesis testing are not applicable.

## Discussion

This pilot study will represent an important step toward developing an accessible, culturally tailored YT program for HSCT patients across diverse international settings. Despite growing interest in integrative oncology, patients are rarely engaged in the design or cultural adaptation of interventions, regardless of setting. This trial seeks to address that gap by involving patients directly in developing a YT program, while also examining how such interventions can be meaningfully adapted and implemented in LMICs such as India and Tanzania.

The primary objective is to develop a culturally tailored YT program that addresses the need for culturally appropriate language, beliefs, and delivery formats that resonate with patients’ lived experiences. By conducting in-depth, semi-structured interviews with HSCT survivors at each site, we will capture nuanced insights about the symptom burden, challenges of HSCT, perception of yoga, psychosocial needs, and barriers to engagement. This information will be critical in refining both content and delivery to ensure relevance and acceptability.

The secondary objective focuses on optimizing the intervention and evaluating its feasibility in an inpatient setting. Offering yoga over a structured timeline (2 weeks pre-transplant through the first 30 days of hospitalization) provides a unique opportunity to assess its utility during a physically and emotionally demanding period. The inclusion of multiple delivery methods (in-person, telehealth, or hybrid) also allows for flexibility and broader applicability in settings with varying levels of infrastructure. Feasibility will be evaluated through adherence to yoga sessions and completion of follow-up assessments, with predefined thresholds to determine whether this intervention can be scaled for future trials. Furthermore, semi-structured exit interviews will offer patient feedback on intervention components and guide further modifications for a larger RCT.

A major strength of this protocol lies in its cultural tailoring, achieved through patient-centered design across 3 geographically and socioeconomically distinct sites. The multicenter international collaboration will lead to intervention’s adaptability but also helps build local capacity, particularly in low-resource settings such as MNH in Tanzania. The flexible delivery model (in-person, virtual, or hybrid) is another asset, increasing the intervention’s feasibility and reach across various contexts. Additionally, the integration of yoga therapy during the inpatient treatment window enhances engagement while minimizing external variability.

As a pilot study, there are inherent limitations to having a small sample size and the lack of a control group. However, this is a common design appropriate for exploring feasibility and cultural adaptation. There is also potential for selection bias, as patients who choose to enroll may already be inclined toward or familiar with yoga. Despite translated materials, language and literacy barriers may persist, particularly in LMICs. Finally, technology access for virtual sessions remains a concern, especially in regions with unreliable connectivity or limited digital literacy.

Overall, this study is expected to yield essential data on cultural adaptability and the feasibility of yoga in inpatient HSCT, laying the groundwork for a larger RCT. Findings from this work may help establish YT as an evidence-informed, patient-centered, and globally scalable supportive care modality for HSCT patients.

## Supplemental Material

Supplemental material - Cultural Adaptation and Feasibility of an Inpatient Yoga Intervention for Patients Undergoing Hematopoietic Stem Cell Transplantation in Tanzania, India, and the United States: A Study Protocol Supplemental material for Cultural Adaptation and Feasibility of an Inpatient Yoga Intervention for Patients Undergoing Hematopoietic Stem Cell Transplantation in Tanzania, India, and the United States: A Study Protocol by Smitha Mallaiah, MSc, C-IAYT, Amanda L Olson, MD, May Daher, MD, Scherezade K. Mama, Dr.PH, Santhosshi Narayanan, MD, Nagarathna R, MD, Raghavendra Rao, PhD, Sachin Jadhav, MD, Nataraj KS, MD, Vijaya Majumdar PhD, Stella Rwezaula, MD, Peter Muhoka, MD, Manjunath NK PhD, Lorenzo Cohen, PhD in Global Advances in Integrative Medicine and Health

Supplemental material - Cultural Adaptation and Feasibility of an Inpatient Yoga Intervention for Patients Undergoing Hematopoietic Stem Cell Transplantation in Tanzania, India, and the United States: A Study Protocol Supplemental material for Cultural Adaptation and Feasibility of an Inpatient Yoga Intervention for Patients Undergoing Hematopoietic Stem Cell Transplantation in Tanzania, India, and the United States: A Study Protocol by Smitha Mallaiah, MSc, C-IAYT, Amanda L Olson, MD, May Daher, MD, Scherezade K. Mama, Dr.PH, Santhosshi Narayanan, MD, Nagarathna R, MD, Raghavendra Rao, PhD, Sachin Jadhav, MD, Nataraj KS, MD, Vijaya Majumdar PhD, Stella Rwezaula, MD, Peter Muhoka, MD, Manjunath NK PhD, Lorenzo Cohen, PhD in Global Advances in Integrative Medicine and Health

Supplemental material - Cultural Adaptation and Feasibility of an Inpatient Yoga Intervention for Patients Undergoing Hematopoietic Stem Cell Transplantation in Tanzania, India, and the United States: A Study Protocol Supplemental material for Cultural Adaptation and Feasibility of an Inpatient Yoga Intervention for Patients Undergoing Hematopoietic Stem Cell Transplantation in Tanzania, India, and the United States: A Study Protocol by Smitha Mallaiah, MSc, C-IAYT, Amanda L Olson, MD, May Daher, MD, Scherezade K. Mama, Dr.PH, Santhosshi Narayanan, MD, Nagarathna R, MD, Raghavendra Rao, PhD, Sachin Jadhav, MD, Nataraj KS, MD, Vijaya Majumdar PhD, Stella Rwezaula, MD, Peter Muhoka, MD, Manjunath NK PhD, Lorenzo Cohen, PhD in Global Advances in Integrative Medicine and Health

Supplemental material - Cultural Adaptation and Feasibility of an Inpatient Yoga Intervention for Patients Undergoing Hematopoietic Stem Cell Transplantation in Tanzania, India, and the United States: A Study Protocol Supplemental material for Cultural Adaptation and Feasibility of an Inpatient Yoga Intervention for Patients Undergoing Hematopoietic Stem Cell Transplantation in Tanzania, India, and the United States: A Study Protocol by Smitha Mallaiah, MSc, C-IAYT, Amanda L Olson, MD, May Daher, MD, Scherezade K. Mama, Dr.PH, Santhosshi Narayanan, MD, Nagarathna R, MD, Raghavendra Rao, PhD, Sachin Jadhav, MD, Nataraj KS, MD, Vijaya Majumdar PhD, Stella Rwezaula, MD, Peter Muhoka, MD, Manjunath NK PhD, Lorenzo Cohen, PhD in Global Advances in Integrative Medicine and Health.

## Data Availability

No datasets were generated or analyzed during the current study. All relevant data from this study will be made available upon study completion.*
