# Supplementary material for: Cultural Adaptation and Feasibility of an Inpatient Yoga Intervention for Patients Undergoing Hematopoietic Stem Cell Transplantation in Tanzania, India, and the United States: A Study Protocol
Source: Glob Adv Integr Med Health. 2026 Apr 1;15:27536130261440941. doi: 10.1177/27536130261440941 (PMC13049346; doi:10.1177/27536130261440941)
Supplement: Supplemental material - Cultural Adaptation and Feasibility of an Inpatient Yoga Intervention for Patients Undergoing Hematopoietic Stem Cell Transplantation in Tanzania, India, and the United States: A Study Protocol [file sj-pdf-2-gam-10.1177_27536130261440941.pdf]

Informed Consent/Authorization for Participation in Research

Title of Research Study: Cultural tailoring and pilot testing of an inpatient yoga therapy program for cancer patients undergoing hematopoietic stem cell transplantation in India, Tanzania, and the United States

Study Number: 2022-0785 – Part 2

Principal Investigator: Dr. Lorenzo Cohen

______________________________ ______________________________
Participant's Name Medical Record Number or Study ID

Key Information

The following is a short summary of this study to help you decide whether or not to be a part of this study. More detailed information is listed later on in this form.

Why am I being invited to take part in a research study?

You are invited to take part in a research study because you are a cancer patient and are scheduled to undergo a hematopoietic stem cell transplantation (HSCT).

What should I know about a research study?

- Someone will explain this research study to you.
- Whether or not you take part is up to you.
- You can choose not to take part.
- You can agree to take part and later change your mind.
- Your decision will not be held against you.
- You can ask all the questions you want before you decide.

Why is this research being done?

The goal of this research study is to develop a yoga program for patients undergoing HSCT.

Yoga combines gentle movements, breathing exercises, relaxation techniques, and

meditation all tailored to the participant's needs. Yoga has no connection to a specific religion or spiritual practice and is intended to align with each person's personal belief system.

Researchers want to use the information learned in this study to see if the yoga program helps to improve patient quality of life.

How long will the research last, and what will I need to do?

As part of this study, you will participate in 12 yoga sessions, complete 4 questionnaires, and be asked to participate in a one-on-one exit interview with the study team. Your participation in this study may last up to 2 ½ months.

More detailed information about the study procedures can be found under ***"What happens if I agree to be in this research?"***

Is there any way being in this study could be bad for me?

Questionnaires may contain questions that are sensitive in nature. You may refuse to answer any question that makes you feel uncomfortable.

You may not like the yoga program. If you are uncomfortable, you may leave the study at any time.

More detailed information about the risks of this study can be found under ***"Is there any way being in this study could be bad for me? (Detailed Risks)"***

Will being in this study help me in any way?

There may be no benefits to you from your taking part in this research. It cannot be promised that there will be any benefits to others from your taking part in this research. However, this research may give healthcare providers a better understanding of how to improve clinical outcomes for cancer patients in the future.

What happens if I do not want to be in this research?

Participation in research is completely voluntary. You can decide to participate, not participate, or discontinue participation at any time without penalty or loss of your regular benefits.

Your alternative to participating in this research study is to not participate.

Detailed Information

The following is more detailed information about this study in addition to the information listed above.

Who can I talk to if I have questions or concerns?

If you have questions, concerns, or complaints, or think the research has hurt you, talk to the study doctor, Dr. Lorenzo Cohen at 713-745-4260, or the Integrative Medicine Research Program Admin Director, Jewel Ochoa at 713-563-4008.

This research has been reviewed and approved by an Institutional Review Board ("IRB" - an ethics committee that reviews research studies). You may talk to them at (713) 792-6477 or IRB_Help@mdanderson.org if:

- Your questions, concerns, or complaints are not being answered by the research team.
- You cannot reach the research team.
- You want to talk to someone besides the research team.
- You have questions about your rights as a research participant.
- You want to get information or provide input about this research.

How many people will be in this study?

It is expected about 15 people at MD Anderson will be enrolled in this part of this research study. It is expected that about 45 people (15 patients from each location in the U.S., India, and Tanzania) will be enrolled in this part of this research study.

What happens if I agree to be in this research?

Screening Questions
Signing this consent form does not mean that you will be able to take part in this study. To help the doctor decide if you are eligible to take part in this study, you will be asked questions about your diagnosis, physical and mental condition, and treatment plan. This should take about 5 minutes to complete and may be done in person, by phone, or by video call (using FaceTime, Skype, or other approved video call methods).

If the screening questions show that you are not eligible to take part in the study, you will not be enrolled.

Questionnaires
If you are found to be eligible and agree to take part in the study, information about you (such as age, gender, race, ethnicity, marital status, education, employment status, religious beliefs, and information about your cancer diagnosis and history, current therapy, and goal of cancer therapy) will be collected. You will complete 4 questionnaires

about any symptoms you may be having, any anxiety and/or depression you may be having, your quality of life, and your expectations about your treatment. It should take about 20-30 minutes to complete all 4 questionnaires. These will be done before your transplant, and then 15 days, 2 months, and 4 months after the transplant.

The questionnaires may be done in person with the help of your caregiver (if needed), by phone, by email, or by text. If you choose to complete the questionnaires through email or text, you will receive a link containing the questionnaires.

Yoga Sessions
You will have up to 12 sessions, 2-3 sessions per week, starting 2 weeks before the transplant and over the first 30 days of your hospitalization. During these sessions, you will be taught about meditation techniques (such as deep-breathing exercises) and gentle movements. You will practice these techniques for about 1 hour every day. You may experience sore muscles after the yoga sessions. You will be taught to only move within a range where you feel comfortable. The yoga sessions will be audio- and video-recorded. The audio and video recordings will be stored for up to 10 years on institutionally approved devices/servers.

You will also be asked to log when you do your yoga practice and your mood and symptoms before and after the session. You will do this throughout the study, and it should take you about 5-10 minutes to complete. You will be given a yoga mat to use for these exercises.

Exit Interview
The study staff may also interview you about your participation in the study. The interview will last about 30-45 minutes. The interview will be conducted over the phone or remotely over Zoom, FaceTime, or other approved video call methods.). The interview will be audio and video recorded and transcribed (typed).

What happens if I say yes, but change my mind later?

You can leave the research at any time; it will not be held against you.

If you withdraw from this study, your medical care and treatments will not be affected.

If you stop being in the research, any data that has already been collected may not be removed from the study database. However, all the data will be de-identified and unable to be traced back to you.

Is there any way being in this study could be bad for me? (Detailed Risks)

Questionnaires and interviews may contain questions that are sensitive in nature. You

may refuse to answer any question that makes you feel uncomfortable. If you have concerns about completing the questionnaire and/or interview, you are encouraged to contact your doctor or the study chair.

If you are feeling distressed and the study staff or doctor thinks it is needed, you will be referred to another doctor or therapist for additional help.

Yoga practice may result in sore or pulled muscles, physical discomfort, and/or accidental injuries such as falling. You may also experience some physical discomfort such as increased heart rate, shortness of breath, and/or fatigue. You will be taught to only move within a range where you feel comfortable. The breathing exercises may cause dizziness. To avoid any discomfort when breathing, you will be taught to do the breathing pattern at a level where you are comfortable.

Although every effort will be made to keep **study data** safe, there is a chance that your personal health information could be lost or stolen, which may result in a **loss of confidentiality**. All study data will be stored in password-protected computers and/or locked file cabinets during the study and will continue to be stored securely after the study. Only authorized people who are working on this study will have access to study data.

In addition to these risks, this research may hurt you in ways that are unknown.

You will be told about any new information that may affect your health, welfare, or choice to stay in the research.

Will it cost anything to be in this study? Will I be paid to be in this study?

There is no cost to you for taking part in this study. No compensation will be provided for participating in this study. You will be able to keep the yoga mat.

What happens to the information collected for the research?

Efforts will be made to limit the use and disclosure of your personal information, including research study and medical records, to people who need to review this information. Complete secrecy cannot be promised. Organizations that may inspect and copy your information include the IRB and other representatives of this organization.

A participant study number will be assigned to you once you have been enrolled in the study. This participant study number will be used to identify your data in the study report and when reporting any data from the study.

Any personal information that could identify you will be removed or changed before data are shared with other researchers or results are made public.

This research is covered by a Certificate of Confidentiality from the National Institutes of Health. This means that the researchers cannot release or use information, documents, or samples that may identify you in any action or suit unless you say it is okay. They also cannot provide them as evidence unless you have agreed. This protection includes federal, state, or local civil, criminal, administrative, legislative, or other proceedings. An example would be a court subpoena.

There are some important things that you need to know. The Certificate DOES NOT stop reporting that federal, state or local laws require. Some examples are laws that require reporting of child or elder abuse, some communicable diseases, and threats to harm yourself or others. The Certificate CANNOT BE USED to stop a sponsoring United States federal or state government agency from checking records or evaluating programs. The Certificate DOES NOT stop disclosures required by the federal Food and Drug Administration (FDA). The Certificate also DOES NOT prevent your information from being used for other research if allowed by federal regulations.

Researchers may release information about you when you say it is okay. For example, you may give them permission to release information to insurers, medical providers or any other persons not connected with the research. The Certificate of Confidentiality does not stop you from willingly releasing information about your involvement in this research. It also does not prevent you from having access to your own information.

Federal law provides additional protections for your medical records and related health information. These are described below.

Will my data be used for future research?

Your personal information is being collected as part of this study. These data may be used by researchers at MD Anderson, Swami Vivekananda Yoga Anusandhana Samsthana (S-VYASA), Healthcare Global Enterprises (HCG), Muhimbili National Hospital (MNH), or shared with other researchers and/or institutions for use in future research.

In some cases, all of your identifying information may not be removed before your data is used for future research. If future research is performed at MD Anderson, the researchers must get approval from the MD Anderson IRB before your data can be used. At that time, the IRB will decide whether or not further permission from you is required. If this research is not performed at MD Anderson, MD Anderson will not have oversight of any data.

What else do I need to know?

This research is being funded by the National Cancer Institute (NCI)

MD Anderson may benefit from your participation and/or what is learned in this study.

Your information and samples (both identifiable and de-identified) may be used to create products or to deliver services, including some that may be sold and/or make money for others. If this happens, there are no plans to tell you, or to pay you, or to give any compensation to you or your family.

Authorization for Use and Disclosure of Protected Health Information (PHI):

A. During the course of this study, MD Anderson will be collecting and using your PHI, including identifying information, information from your medical record, and study results. For legal, ethical, research, and safety-related reasons, your doctor and the research team may share your PHI with:
   - The Office for Human Research Protections (OHRP)
   - The IRB and officials of MD Anderson
   - The National Cancer Institute (NCI), who is a sponsor or supporter of this study, and/or any future sponsors/supporters of the study, and/or licensees of the study technology
   - Study monitors and auditors who verify the accuracy of the information
   - Individuals who put all the study information together in report form

   Study sponsors and/or supporters receive limited amounts of PHI. They may also view additional PHI in study records during the monitoring process. MD Anderson's contracts require sponsors/supporters to protect this information and limit how they may use it.

   The results of this research may be published in scientific journals or presented at medical meetings, but your identity will not be disclosed.

B. Signing this consent and authorization form is optional but you cannot take part in this study or receive study-related treatment if you do not agree and sign.

C. MD Anderson will keep your PHI confidential when possible (according to state and federal law).

   Once disclosed outside of MD Anderson, federal privacy laws may no longer protect your PHI.

D. The permission to use your PHI will continue indefinitely unless you withdraw your authorization in writing. Instructions on how to do this can be found in the MD Anderson Notice of Privacy Practices (NPP) or you may contact the Chief Privacy Officer at 713-745-6636. If you withdraw your authorization, you will be removed from the study and the data collected about you up to that point can be used and included in data analysis. However, no further information about you will be collected.

CONSENT/AUTHORIZATION

I understand the information in this consent form. I have had a chance to read the consent form for this study, or have had it read to me. I have had a chance to think about it, ask questions, and talk about it with others as needed. I give the study chair permission to enroll me on this study. By signing this consent form, I am not giving up any of my legal rights. I will be given a signed copy of this consent document.

_______________________________________ ________
SIGNATURE OF PARTICIPANT DATE

____
PRINTED NAME OF PARTICIPANT

PERSON OBTAINING CONSENT
I have discussed this research study with the participant and/or his or her authorized representative, using language that is understandable and appropriate. I believe that I have fully informed this participant of the nature of this study and its possible benefits and risks and that the participant understood this explanation.

____ __________
PERSON OBTAINING CONSENT DATE

____
PRINTED NAME OF PERSON OBTAINING CONSENT
