# Supplementary material for: Cultural Adaptation and Feasibility of an Inpatient Yoga Intervention for Patients Undergoing Hematopoietic Stem Cell Transplantation in Tanzania, India, and the United States: A Study Protocol
Source: Glob Adv Integr Med Health. 2026 Apr 1;15:27536130261440941. doi: 10.1177/27536130261440941 (PMC13049346; doi:10.1177/27536130261440941)
Supplement: Supplemental material - Cultural Adaptation and Feasibility of an Inpatient Yoga Intervention for Patients Undergoing Hematopoietic Stem Cell Transplantation in Tanzania, India, and the United States: A Study Protocol [file sj-pdf-3-gam-10.1177_27536130261440941.pdf]

Semi Structured Interview guide

Hi[Greeting]!

My name is [insert name], and I am currently a [insert position/role] at [institution]. We are conducting a study to develop a culturally tailored yoga therapy program for patients receiving Hematopoietic stem cell transplantation (HSCT). We are trying to understand program preferences and examine cultural factors related to yoga and psychosocial support.

Thank you for agreeing to participate in this interview, which will help inform the development of the Yoga therapy program for cancer patients. I will record our interview and take notes as we speak.

Start recording

Please answer the questions as best you can and know that we can skip any questions you do not want to answer. Before we start, may I use your first name during the interview, or would you prefer me to call you by a different name?

Preferred name___________

Do you have any questions before we get started?

HSCT experience and symptom burden

I will begin the interview by asking you about your Hematopoietic Stem Cell Transplantation (HSCT) experience and the symptoms you have had during the hospital stay.

1. Tell me about the most challenging symptoms you experienced post-HSCT.
2. What was the most challenging part of your HSCT?
    a. Probe for challenges related to the procedure, symptom burden, isolation, etc.
3. If you could choose to manage one acute symptom, what would that be?
4. Can you describe how you coped with physical and emotional challenges related to your cancer and HSCT treatment? (If needed, probe for simple practices in their lifestyle, like exercise, prayer, etc.)

Yoga Background

5. Describe what you have heard about yoga and your thoughts about yoga.
6. Have you tried any form of yoga before?

a. If yes, ask the participant to share more about their yoga experience.

b. If no, skip to the next question.

7. Describe for me what you know about meditation.

8. Have you ever tried meditation?

a. If yes, ask the participant to tell you more about their experience with meditation.

b. If no, skip to the next question.

Yoga Description

I want to describe what yoga is in our study and what our yoga therapy program may look like with examples.

Yoga is a group of physical, mental, and spiritual practices or disciplines that originated in ancient India and aim to unify the mind and body. It involves various practices like movement, meditation, and breathing techniques to promote mental and physical well-being. Yoga is recommended for managing various symptoms of cancer treatment and can be used during and after cancer treatment. The following four components will comprise the Yoga program: 1) joint loosening and stretching exercises; 2) Different yogic postures and a deep relaxation technique; 3) Pranayama/regulated nostril breathing and breath energization techniques; and 4) meditation.

Example 1 Demonstration video/picture of Sukshma Vyayama – Joint loosening exercises

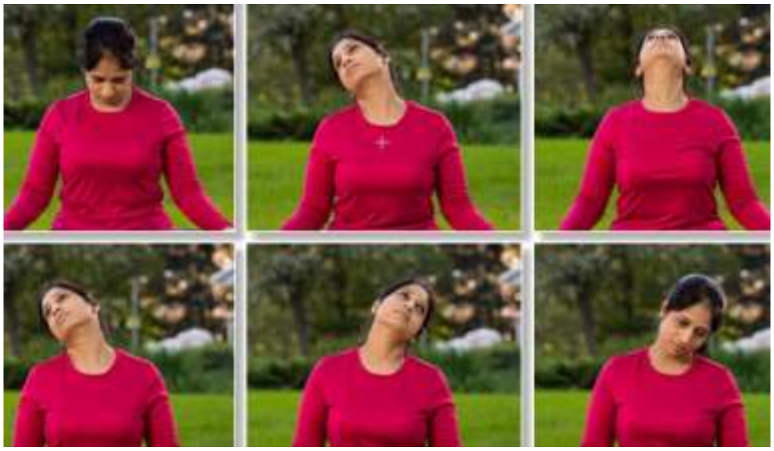

Example 2 Demonstration video/picture of relaxation and meditation

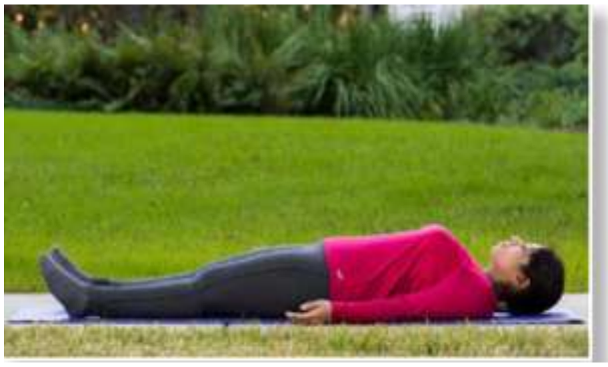
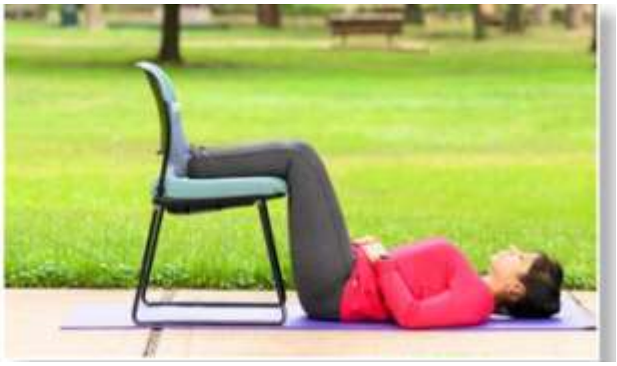
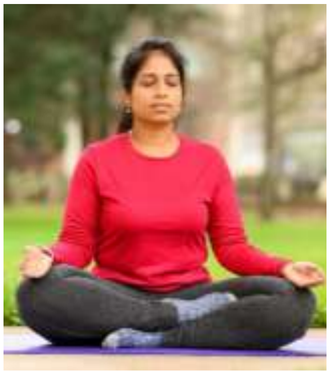
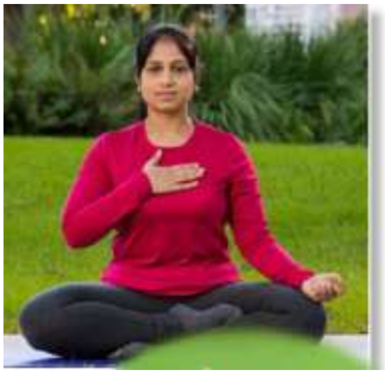
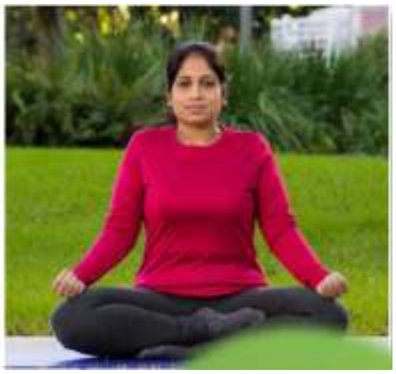

Intervention

One of the goals of this study is to gather information that will help us design a Yoga therapy program tailored to address the needs of cancer patients receiving HSCT at [location]. Now that you have an idea of what this program may look like and you know what it is like to have an HSCT, I would like to get some information on what you think your preferences would be if you had participated in this yoga program during the first 30 days of your HSCT when you were in the hospital.

The following few questions will focus on the multi-component yoga program incorporating gentle stretching, relaxation, breathing, and meditation that we want to test.

9. If you were going to participate in a yoga program, from whom would you want to hear about the program? probe for the doctor, nurse, or research support staff
10. Tell me about which aspects of the program – physical movement and relaxation/meditation – you think you would like the most and why.
11. How do you think the physical movement component would have helped you during the transplant process and hospitalization?
12. What barriers do you think you would have had to participating in the physical movement component?

13. How do you think the relaxation/meditation component would have helped you during the transplant process and hospitalization?
14. What barriers do you think you would have had to participating in the relaxation/meditation component?
15. Tell me how important it is to you to know about the usefulness/benefits of these yoga practices during the intervention.
    Probe for information upfront, during, or later.

The number of sessions/doses:

We are determining the ideal number of sessions to offer participants and the ideal length of time for the sessions. If you were participating in a yoga program in the first 30 days after HSCT:

16. What is the ideal number of sessions per week? [Daily, a few times a week?]
17. What is the ideal session length? [30 mins, 45 mins, 60 mins or longer]
18. What is the ideal time for the sessions – mid-morning or afternoon for your yoga classes?
19. Daily, how much time do you think you could have dedicated to self-practice if you had all the supporting materials provided?
20. What mode of teaching would you prefer? Probe for in-person, online (zoom), or hybrid
21. Do you have a language preference for receiving instruction or materials? If yes, please specify the language.

Cultural adaptations/preferences

We are trying to understand any perceived cultural barriers to receiving and following the yoga program.

22. What are some of the characteristics you would expect in a Yoga therapist?
23. What characteristics would you expect in a Yoga Model for education materials in terms of photos, etc.?
24. In general, what other concerns do you have about the yoga program and if you were doing the yoga program before and during your hospitalization for HSCT?
25. Based on your experience as a cancer patient and survivor, what barriers that we haven't discussed do you see for engaging in a yoga program before and during your hospitalization for HSCT?

26. What are some potential solutions to the barriers you described that would help more cancer patients participate in a yoga program that is offered at the place where they receive their cancer treatment?

Turn off the recorder.

Thank you for contributing to this study and for your time. If you have any questions in the future, please do not hesitate to contact our research team
