# Supplementary material for: Cultural Adaptation and Feasibility of an Inpatient Yoga Intervention for Patients Undergoing Hematopoietic Stem Cell Transplantation in Tanzania, India, and the United States: A Study Protocol
Source: Glob Adv Integr Med Health. 2026 Apr 1;15:27536130261440941. doi: 10.1177/27536130261440941 (PMC13049346; doi:10.1177/27536130261440941)
Supplement: Supplemental material - Cultural Adaptation and Feasibility of an Inpatient Yoga Intervention for Patients Undergoing Hematopoietic Stem Cell Transplantation in Tanzania, India, and the United States: A Study Protocol [file sj-pdf-4-gam-10.1177_27536130261440941.pdf]

EXIT INTERVIEW

This is a semi-structured interview with open dialogue encouraged. Interviews will be recorded and transcribed.

GENERAL

Thank you for agreeing to this interview and to having it recorded.

1. Can you describe your overall experience with the Yoga Therapy for Stem Cell Transplant research study?

PRIOR EXPOSURE TO YOGA, YOGA THERAPY, AND OTHER MIND-BODY PRACTICES

2. What, if any, was your exposure to yoga before participating in this research study?

3. Before this study, have you ever done mind-body practice such as yoga, meditation, or others (qigong or tai chi)? Could you elaborate on that experience

CONSENT

4. What were the primary reasons you were interested in consenting to this study?

5. When considering consenting to the study, what were the primary factors that helped you decide to participate?

6. Were there any aspects of the study description you were provided that might have discouraged you from participating?

7. What role, if any, did your clinical team (doctors, nurses) have in your decision to participate in this study?

8. Having completed the study, what recommendations or advice would you give our coordinators approaching potential patients to the study? (any gaps?)

9. Some potential participants may express uncertainty about this study when it is first discussed with them – What recommendations do you have for future participants during the consent process

10. Is there any other comment you would like to make regarding your experience consenting to the study that we didn't cover?

ELECTRONIC MATERIALS

One important aspect of this research study is technology, as we are assessing the feasibility of remote consenting and electronic data collection programs.

11. How would you prefer to complete the questionnaires: remotely (via email) or in person? Please explain your preference.

12. In what ways, if any, did you find it helpful to consent remotely and have questionnaires sent to you electronically via email?

13. Did your coordinators help you with any issues regarding the electronically delivered questionnaires? Did you experience any glitches or technical difficulties at all?

14. Do you feel you were given adequate instructions regarding the electronically delivered questionnaires?

15. What was the best or most helpful about the electronic questionnaires?

16. What was the worst or least helpful thing about the electronically delivered questionnaires?

17. Did any of the questionnaire items make you feel uncomfortable, or were there any you chose not to answer? Do you remember which ones?

THE STUDY

Intervention:

18. Please tell me about your experience with the yoga sessions you had before your transplant.

19. Please tell me about your experience with the yoga sessions after your transplant.

20. Did you have yoga sessions after being discharged from the hospital? If so, tell me about your experience with these sessions after you left the hospital.

21. Can you share about your experience about in person vs virtual yoga sessions?

22. Did you feel you could communicate well with your yoga therapist?

23. How did you feel about the pace of your yoga sessions? Too fast or too slow?

24. Did you practice yoga outside the class? How did it help?

25. Would you change anything about how your yoga therapy sessions were managed?

Questionnaire collection:

26. Did you feel you could communicate with the study coordinator?

27. How did you feel about the timing of your study questionnaires?

28. Is there anything you would change about the study questionnaires?
29. What was your favorite aspect of this study?
30. What was one thing that could have been improved to enhance your experience?

POST-STUDY

31. Did participation in this program inspire you to explore yoga practices further? Tell me more about it
32. Would you recommend yoga therapy to others preparing for a similar transplant?

FINAL THOUGHTS & CLOSING

33. Is there anything we haven't discussed that you think is important for us to know?

Thank you so much for your time, participation, and feedback. If in the future there is anything else you would like to tell us about yoga therapy, the study, or your research team, that would help us improve, please don't hesitate to contact us.
